# Implementation fidelity in a multifaceted program to foster rational antibiotics use in primary care: an observational study

**DOI:** 10.1186/s12874-022-01725-3

**Published:** 2022-09-19

**Authors:** Lukas Kühn, Dorothea Kronsteiner, Petra Kaufmann-Kolle, Edith Andres, Joachim Szecsenyi, Michel Wensing, Regina Poss-Doering

**Affiliations:** 1 Department of General Practice and Health Services Research, University Hospital Heidelberg, Im Neuenheimer Feld 130.3, 69120 Heidelberg, Germany; 2 Institute of Medical Biometry, University of Heidelberg, Im Neuenheimer Feld 130.3, 69120 Heidelberg, Germany; 3 aQua Institut, Maschmuehlenweg 8-10, 37073 Goettingen, Germany

**Keywords:** Complex intervention, Fidelity analysis, Pragmatic trial, Quality circles, Rational antibiotic use

## Abstract

**Background:**

The ARena study (Sustainable Reduction of Antimicrobial Resistance in German Ambulatory Care) is a three-arm, cluster randomized trial to evaluate a multifaceted implementation program in a German primary care setting. In the context of a prospective process evaluation conducted alongside ARena, this study aimed to document and explore fidelity of the implementation program.

**Methods:**

This observational study is based on data generated in a three-wave survey of 312 participating physicians in the ARena program and attendance documentation. Measures concerned persistence of participation in the ARena program and adherence to intervention components (thematic quality circles, e-learning, basic expenditure reimbursements, additional bonus payments and a computerized decision support system). Participants’ views on five domains of the implementation were also measured. Binary logistic and multiple linear regression analyses were used to explore which views on the implementation were associated with participants’ adherence to quality circles and use of additional bonus compensation.

**Results:**

The analysis of fidelity showed overall high persistence of participation in the intervention components across the three intervention arms (90,1%; 97,9%; 92,9%). 96.4% of planned quality circles were delivered to study participants and, across waves, 30.4% to 93% of practices participated; 56.1% of physicians attended the maximum of four quality circles. 84% of the practices (*n* = 158) with a minimum of one index patient received a performance-based additional bonus payment at least once. In total, bonus compensation was triggered for 51.8% of affected patients. Participation rate for e-learning (a prerequisite for reimbursement of project-related expenditure) covered 90.8% of practices across all intervention arms, with the highest rate in arm II (96.5%). Uptake of expenditure reimbursement was heterogeneous across study arms, with a mean rate of 86.5% (89.1% in arm I, 96.4% in arm II and 74.1% in arm III). Participants’ views regarding participant responsiveness (OR = 2.298) 95% CI [1.598, 3.305] and Context (OR = 2.146) 95% CI [1.135, 4.055] affected additional bonus payment. Participants’ views on participant responsiveness (Beta = 0.718) 95% CI [0.479, 0.957], Context (Beta = 0.323) 95% CI [0.055, 0.590] and Culture of shared decision-making (Beta = -0.334) 95% CI [-0.614, -0.053] affected quality circle attendance.

**Conclusion:**

This study showed an overall high fidelity to the implementation program. Participants’ views on the implementation were associated with degree of intervention fidelity.

**Trial registration:**

ISRCTN, ISRCTN58150046.

**Supplementary Information:**

The online version contains supplementary material available at 10.1186/s12874-022-01725-3.

## Introduction

### Background

Pragmatic trials are applied to inform health policy decision makers about the effectiveness of interventions used in healthcare practice [1]. Formative evaluations of such trials can provide added information to primary and secondary study outcomes since effect sizes alone do not grant sufficient information about the replicability of trial outcomes [2] or the level of implementation fidelity [3]. Findings need to be contextualized to the feasibility of implementation programs [4]. Only if feasibility is high, observed effects can be attributed to the respective program. To understand mechanisms affecting feasibility, investigations on implementation fidelity are inevitable [4]. Potentially, factors affecting implementation fidelity can be explored in qualitative research approaches [5–7], yet this approach does not allow statistical associations and critical consideration of program feasibility regarding study outcomes. Hence, the present study reports findings of a quantitative fidelity analysis conducted alongside a multifaceted pragmatic trial.

This study is based on a three-armed cluster randomized trial (ARena) designed to sustainably reduce antimicrobial resistance in German ambulatory care [8]. In Germany, about 85% of antibiotics used in human medicine are prescribed in ambulatory care [9]. Most common prescription fields are respiratory tract infections which are, contrary to the effects of antibiotics, predominantly of viral origin [10]. Multiple reasons have been identified for such inappropriate prescribing patterns: Physicians report to face diagnostic insecurities, demanding patient expectations and a personal desire to be on the safe side in treatment procedures [11–13]. Since physicians are aware of this matter, 75% of surveyed resident physicians in Germany wish to receive training offers which address a rational use of antibiotics [13]. Previous approaches to foster this rationality included public awareness campaign strategies, financial incentivization of a rational prescription-behaviour, reliable patient information sources, improvement of patient-provider communication and the provision of point of care testing [14–19]. Frequently, a combination of listed interventions promised the highest effects [20]. Nevertheless, relevant data from German ambulatory practices are rare, and findings of implementation programs conducted in other healthcare systems are only partly transferrable to primary care settings in Germany. Besides, a sustained uptake of measures beyond intervention periods could not yet be proven.

The ARena study addressed this gap by providing a standard set of implementation strategies across study arms comprising of e-learning on communication with patients, quality circles (QC) with data-based feedback for physicians, information campaigns for the public, patient information material and performance-based additional bonus compensation. QCs have widely been adopted and participation rates of primary care physicians in Europe increased substantially in the last decades [21–24]. Initially used to support continuous medical education, QCs are nowadays mainly applied for quality improvement purposes [23]. In this respect, QCs intend to foster guideline-oriented prescribing patterns and to support desired change of outdated routines. Yet, effects meeting these targets are heterogeneous within and across studies and cannot be considered thorough yet [25–28].

The performance-based additional compensation in ARena was designed as a bonus payment system similar to Heider & Mang [29] based on antibiotic prescribing. Thus, it needs to be distinguished from pay-for-performance systems where additional reimbursements are paid for reaching predefined thresholds of quality indicators. Systematic reviews addressing bonus payments have been conducted in the context of smoking cessation endeavours [30] and to increase the supply of breast, cervical and colorectal cancer screenings [31]. Both reviews included studies of moderate quality and the inconsistency of results did not permit a conclusion about additional bonus compensations. Research investigating effects on additional bonus compensations regarding a rational use of antibiotics was not identified. The fidelity analysis reported in this study explored the overall participation in the implementation program across all study arms. A particular focus was put on the two key program components of QCs and additional bonus compensation since these were distinctive features in comparison to other research efforts regarding the rational use of antibiotics conducted at the same time in German primary care [32].

### Objective

The aim of this study was to document and explore fidelity to an implementation program embedded in a multifaceted cluster randomized trial in a two-step approach: (1) Description of participants’ engagements to intervention components and perceived influencing domains affecting fidelity; (2) Exploration of the associations between engagement in intervention components and perceptions of influencing domains.

## Methods

### Theoretical conceptualization

**Fig. 1:**
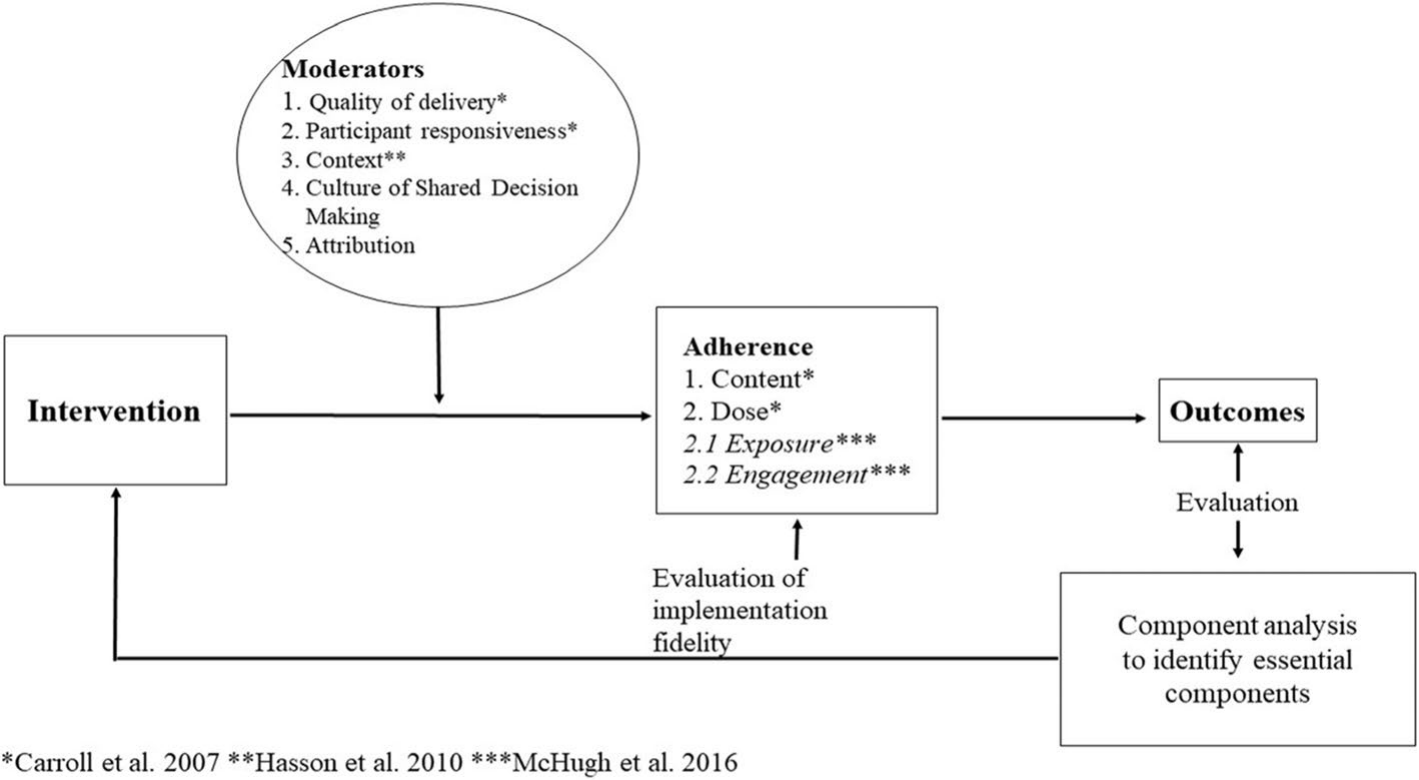
Modified conceptual framework of implementation fidelity

Fidelity as a term describes the level to which an intervention was delivered as intended [4]. In this respect, the most commonly practiced framework [33] distinguishes between adherence and moderator domains to provide a cause-effect-principle explaining fidelity measures. Following this comprehension, adherence is defined as a bottom-line measurement describing the dose and content of an implementation program. If an intervention completely adheres to a study protocol, fidelity can be rated high. To understand mechanisms affecting adherence scales, factors that affect the level of fidelity need to be identified. In this study, these factors originated from five self-reported domains describing participants’ perceived views on implementation. Figure 1 comprises the elements of adherence and considered domains. Since the framework on implementation fidelity has continuously been extended, this analysis included the additional domain of ‘context’ introduced by Hasson [34]. The characterization of dose in quality improvement measurements provided by McHugh et al. [35] was included into the theoretical model for this present study.

### Study design of the ARena trial

The ARena implementation program was designed as a three-armed, non-blinded cluster randomized trial with an added cohort reflecting standard care. Randomization was performed by the Institute of Medical Biometry at the University Hospital Heidelberg. The implementation program was organized by the aQua Institut, Goettingen, and embedded into 14 primary care networks (PCN) in two federal states (Bavaria and North Rhine-Westphalia) in Germany. PCNs are regional associations of primary care practices aiming at facilitating quality improvement initiatives, representing interests at health insurance companies as well as reimbursing additional activities for member practices [36]. In order to understand the role of PCNs in the dissemination of the implementation program, this level of randomization has been chosen for primary outcome analysis. The implementation program consisted of different components applied to each of the three study arms. Arm I received a standard set comprising a public information campaign, patient information material, e-learning addressing physician–patient communication, thematically relevant QCs (common respiratory tract infections (CRTI), urinary tract infections (UTI), community acquired pneumonia (CAP), multi-resistant pathogens (MRP)) containing data-based feedback for physicians, and the performance-based bonus. Arm II received the standard set plus e-learning modules addressing patient communication and QCs targeting non-physician health professionals as well as patient information material provided via tablet devices. Arm III received the standard set, a computerized decision support system integrated in existing practice management software and multidisciplinary QCs in local groups. All participating practices could receive reimbursement for project-related expenditure. A detailed display of the study design is provided by the study protocol [8].

The intervention period encompassed 21 months. In total, 196 practices with 312 physicians and 99 medical assistants (MA) participated. The statutory health insurer AOK (Public organization of statutory health insurance) provided routinely collected claims data referring to consultations for non-complicated infections in the intervention arms and the added cohort reflecting standard care in Bavaria and North Rhine-Westphalia. The study design and detailed sample size descriptions of the ARena trial are illustrated in Additional File 1, Supplementary Fig. 1. Detailed sociodemographic characteristics of included cases, sample size calculation, information about relevant data protection as well as outcomes of the ARena trial regarding a sustainable reduction of antimicrobial resistance in German ambulatory care have been reported elsewhere [37, 38]. All routinely collected claims data relevant for the ARena trial were stored on secure servers at the aQua institute, Göttingen, Germany and were analyzed by a qualified statistitian.

### Study design of the process evaluation

**Fig. 2:**
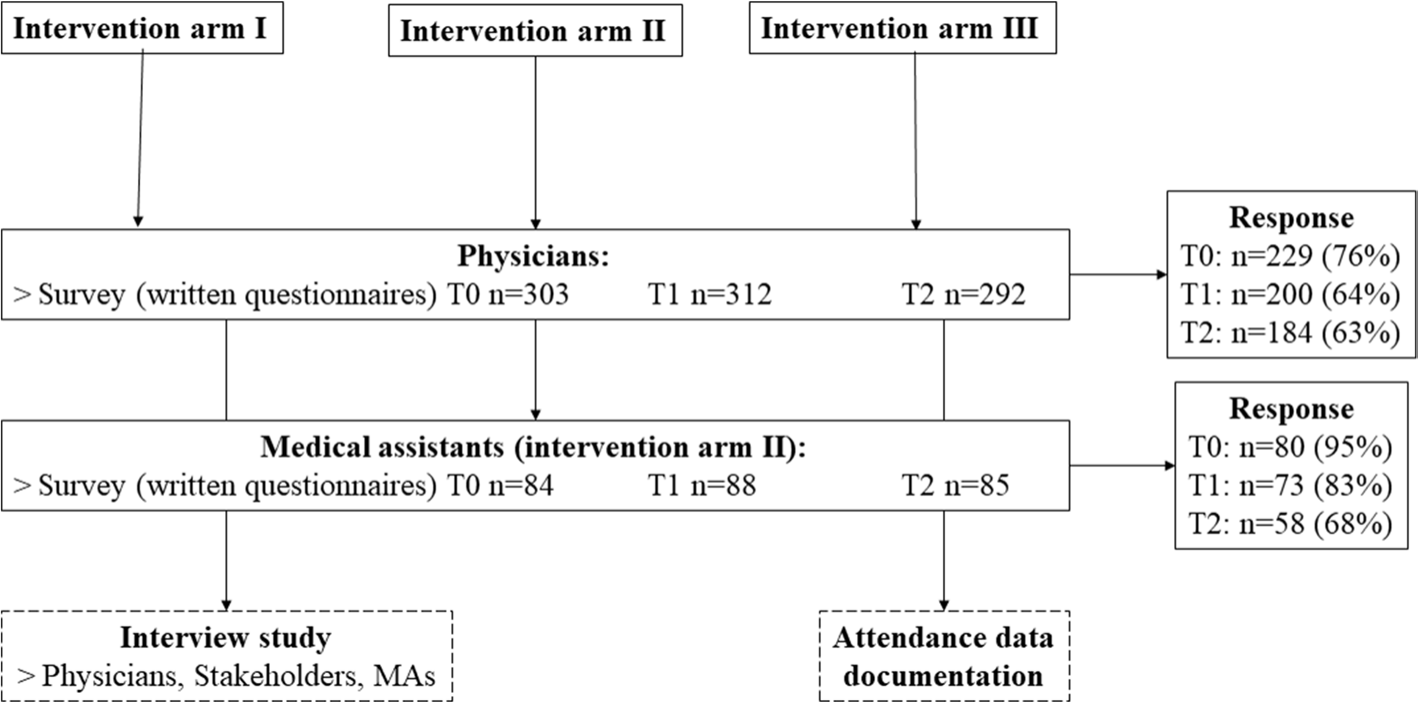
Study design and number of participants in the process evaluation

The ARena trial was accompanied by a process evaluation (PE) which intended to understand working mechanisms affecting primary and secondary outcomes as well as determining the level of fidelity to the program [8]. The PE was designed as a prospective observational study and conducted with a mixed methods approach containing a longitudinal survey study and an interview study. The survey study consisted of written questionnaires targeting participating physicians of study arms I, II and III and participating MAs of study arm II. For each intervention arm, a tailored questionnaire was developed. Data collection took place at three different points in time (T0-T2). The interview study targeted participating physicians, MAs and stakeholder representatives of PCN managements, health insurance providers, the association of statutory health insurance physicians and self-help organisations. Additionally, implementers documented overall participation over the course of the study, utilization of e-learning and computerized decision-support-system (CDSS), attendance to QCs, and reimbursement for project- and patient-related expenditures. This present study was based on survey data collected during the PE and the additional documentation (attendance data). Findings of the PE analyses have been reported elsewhere [39–41]. Figure 2 summarizes the study design and sample size of the PE.

### Study population

An extensive description of the study population of the ARena trial is provided in the protocol [8]. To be eligible for participation in the PE, practices needed to be enrolled in one of the 14 participating PCNs and had to be allocated to one of the three intervention arms. Physicians had to represent one of the medical specialist groups of general practitioners, internists, gynecologists, ear-nose-throat specialists, urologists, pulmonary specialists or pediatricians. MAs eligible for participation in the PE were employees of participating practices. Across participant groups, further inclusion criteria were written and spoken German language skills, 18 years of age or older and a written declaration of consent to participate in the study. No additional exclusion criteria were assigned.

### Recruitment and sampling for the survey study

The PE followed a voluntary response sampling strategy. By signing the consent form of the ARena trial, participants also consented into participating in the PE. The Department of General Practice and Health Services Research at the University Hospital Heidelberg compiled a cover letter and written information material detailing the procedures and aim of the PE. The Department’s ARena study team of researchers developed the study-specific survey questionnaires based on the Theory of Planned Behaviour [42]. The questionnaire items were used to gain insights into the impact of the intervention components and contextual factors [8]. The aQua Institut (Goettingen) led the project and thus contacted enrolled practices and sent the survey questionnaires by mail. After four weeks, e-mail reminders were sent to increase response rates.

### Data collection and measures

#### Survey data

Participants’ views on the implementation and the engagement in key components of ARena (QCs and additional bonus compensation) were explored in a self-reported questionnaire. Questionnaires were dispatched to participants in January 2018 (T0), October 2018 (T1) and July 2019 (T2). All questionnaires focused on adherence to intervention components and views on the implementation. T1 and T2 questionnaires additionally asked for intermediate and final conclusion regarding the assessment of intervention components. Completed questionnaires were returned and registered by the ARena study team at the Department of General Practice and Health Services Research, University Hospital Heidelberg, between February and April 2018 (T0), November 2018 to January 2019 (T1) and July to September 2019 (T2). Received questionnaires were digitalized and transferred into IBM SPSS Statistics 24. Survey items included for each domain of participant views are listed in Additional File 2, Supplementary Table 1.

The participants’ views on the implementation were measured in five domains: 1) ‘Participant responsiveness’ contained items regarding the respondents’ perceptions about the usefulness of components and their potential to facilitate new impulses in the context of rational antibiotics use. 2) ‘Quality of delivery improvement’ referred to reflections about the extent to which the ARena participation supported guideline-oriented prescribing patterns and fostered security in therapeutic decisions. 3) ‘Contextual facilitators’ referred to the role of PCNs in optimizing patient care as they were seen as a major design element of the ARena study. 4) ‘Positive antibiotic attributions’ considered physicians’ perceptions about positive ancillary effects of antibiotic use such as reduced consultation time. 5) ‘The culture of shared decision-making’ (SDM) score reflected the respondents’ integration of patient- and peer-views into therapeutic decisions.

#### Attendance and use of financial bonus

Adherence to QCs, e-learning, CDSS, and basic expenditure reimbursements were identified by documented attendance data. Triggered additional bonus payment was identified from the claims data. Overall, data of 196 practices were collected by the aQua Institut over the intervention period of 21 months between October 2017 and June 2019. Variables regarding participant attendance of QC meetings were reported on practice level and were collected in the respective events. Attendance data was documented in Microsoft Excel 2019 and subsequently transferred to IBM SPSS Statistics 26. Variables providing information about additional bonus compensation were collected using the claims data aggregated on practice level.

Adherence was subcategorized in the domains of content and dose. Indicators representing content were exclusively collected for the additional bonus compensation component. Indicators representing dose which was further split into domains of exposure and engagement were collected for both, QCs and additional compensation components.

### Statistical analyses

Based on the survey and attendance data, the intervention fidelity was explored. Indicators were developed to map the participants’ engagement in five intervention components. The descriptive analysis explored absolute and relative frequencies on physician and practice level in the intervention arms. Sociodemographic factors, adherence data and participants’ views on implementation were analyzed descriptively. For continuous variables, means, medians, min/max and standard deviations were provided, for categorial and ordinal variables absolute and relative frequencies were reported. Survey items were based on a 5-point-Likert scale ranging from “Strongly Disagree” to “Strongly Agree”. Items representing one domain of participants’ views on implementation were scored using mean value calculations and tested on internal consistency using Cronbach’s Alpha procedures. To explore correlates between variables of interest, binary correlations between dependent variables (engagement in additional bonus compensation; engagement in QC themes) and independent variables (participant responsiveness; quality of delivery; context, culture of SDM; positive AB attribution) were determined by calculating Pearson and Spearman correlation coefficients and guided the variable selection for subsequent regression analyses.

A binary logistic regression model was used to identify directional coherence between the engagement in additional bonus compensation representing the outcome variable and the five domains of participant views on implementation representing predictor variables. A multiple linear regression model was computed regarding association between engagement in the four QC themes reflecting the outcome variable and the five domains of participant views reflecting predictor variables. Predictor variables were considered on a metric scale level and tested on multicollinearity with a set threshold of *r* ≥ 0.7. Missing values were marked accordingly and excluded from analyses. Effect sizes were reported by Odds Ratios *(OR)* and Beta coefficients including 95% confidence intervals. To provide information about data accuracy, confidence intervals, standard errors and the coefficients of determination *R*^*2*^ were listed. All models have been adjusted by age, sex and intervention arm affiliation. Additional multilevel analyses were conducted considering a hierarchical data structure of practices representing the random effect (MIXED and GENLIN estimations). Due to a high loss of cases within data linkage efforts between attendance and survey data, outcome variables of estimation models based on self-reports of the T2 survey only. The level of significance was set at *p* ≤ *0.05*. *P*-values were of explorative nature as a pre-determined statistical power calculation for this analysis was not feasible.

## Results

### Adherence to the ARena implementation program

In total, data from 196 participating practices (312 physicians; 78.2% GPs) were collected. 290 physicians (92.9%) continuously participated in ARena over the intervention period. The drop-out rate of included practices as observed in the process evaluation was 4.1% at the end of the intervention period. Indicators describing fidelity to the ARena implementation program are provided in Additional File 2, Supplementary Table 2.

The analysis showed continuous physician participation in the intervention components across all intervention arms with 90.1% in arm I, 97.9% in arm II and 92.9% in arm III. 96.4% (*n* = 54) of the planned QCs could be delivered to between 30.4% and 93% of participants. The maximum of four QCs was attended by 51.6% of the physicians. Participation rate for the e-learning component was 90.8% across all intervention arms on practice level, with the highest rate in arm II (96.5%). Completing the e-learning component qualified 177 practices for the basic expenditure reimbursement meant to cover project-related additional expenditure. This was claimed fairly heterogeneous with a mean rate of 86.5% (89.1% in arm I, 96.4% in arm II and 74.1% in arm III).. In intervention arm III, 51% of practices utilized the offered computerized decision support (CDSS)). A total of 88.4% of physicians (84% of the practices (*n* = 158)) with a minimum of one index patient received the additional bonus compensation at least one time. The descriptive analysis indicated that fidelity appeared the highest in intervention arm II. Table 1 describes findings regarding continuous participation, utilization of e-learning and CDSS, reimbursement of project-related expenditure and participation in QCs calculated on physician level and findings referring to the additional bonus compensation based on practice level.

**Table 1:** Adherence to intervention components

Intervention arm	I	II	III
**Calculation on physician level (intention-to-treat)**	*n* = 111 physicans (%)	*n* = 94 physicians (%)	*n* = 113 physicians (%)
Continuous participation (physicians)	100 (90.1%)	92 (97.9%)	105 (92.9%)
Participation e-learning	99 (89.2%)	89 (94.7%)	94 (83.2%)
Utilization of CDSS	-	-	62 (54.9%)
Basic expenditure reimbursement—claimed	90 (81.1%)	85 (90.4%)	74 (65.5%)
Expenditure reimbursement II - claimed	-	84 (89.4%)	-
Expenditure reimbursement III - claimed	-	-	43 (38.1%)
QC “Upper respiratory tract infections”, participation	72 (64.9%)	78 (83.0%)	67 (59.3%)
QC- “urinary tract infections”, participation	53 (47.7%)	56 (59.6%)	56 (49.6%)
QC- “Multiresistent pathogens “, participation	43 (38.7%)	57 (60.6%)	24 (21.2%)
QC-„Community-aquired pneumonia “, participation	52 (46.8%)	63 (67.0%)	26 (23.0%)
Additional bonus compensation on practice level^a^	Arm I *n* = 69 practices	Arm II *n* = 57 practices	Arm III *n* = 69 practices
2017q4			
Min.1 index patient	58 of 69 (84.1%)	49 of 57 (86.0%)	45 of 69 (65.2%)
Bonus received	24 of 58 (41.4%)	29 of 49 (59.2%)	15 of 45 (33.3%)
2018q1			
Min.1 index patient	61 of 69 (88.4%)	53 of 57 (93.0%)	55 of 69 (79.7%)
Bonus received	31 of 61 (50.8%)	26 of 53 (49.1%)	27 of 55 (49.1%)
2018q2			
Min.1 index patient	58 of 69 (84.1%)	52 of 57 (91.2%)	56 of 69 (81.2%)
Bonus received	27 of 58 (46.6%)	31 of 52 (59.6%)	30 of 56 (53.6%)
2018q3			
Min.1 index patient	57 of 69 (82.6%)	51 of 57 (89.5%)	56 of 69 (81.2%)
Bonus received	29 of 57 (50.9%)	29 of 51 (56.9%)	33 of 56 (58.9%)
2018q4			
Min.1 index patient	58 of 69 (84.1%)	52 of 57 (91.2%)	58 of 69 (84.1%)
Bonus received	28 of 58 (48.3%)	33 of 52 (63.5%)	28 of 58 (48.3%)
2019q1			
Min.1 index patient	58 of 69 (84.1%)	52 of 57 (91.2%)	57 of 69 (82.6%)
Bonus received	31 of 58 (53.4%)	28 of 52 (53.8%)	30 of 57 (52.6%)
2019q2			
Min.1 index patient	57 of 69 (82.6%)	51 of 57 (89.5%)	53 of 69 (76.8%)
Bonus received	30 of 57 (52,6%)	27 of 51 (52.9%)	28 of 53 (52.8%)

^a^ The performance-based additional bonus compensation is based on claims data and can only be calculated on practice level

### Adherence to key components of the ARena implementation program

**Fig. 3:**
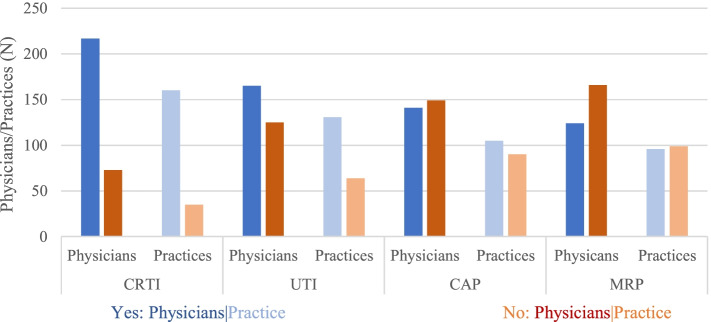
Attendance to QC themes. *Physicians (*N* = 290); practices (*N* = 195)

At this stage, descriptively reported adherence scales represent the entire study sample (Physicians *n* = 290; Practices n = 196). The indicators representing exposure yielded highest scores. In Fig. 3, the number of attendees of all four QC themes are depicted on physician and practice level.

**Fig. 4:**
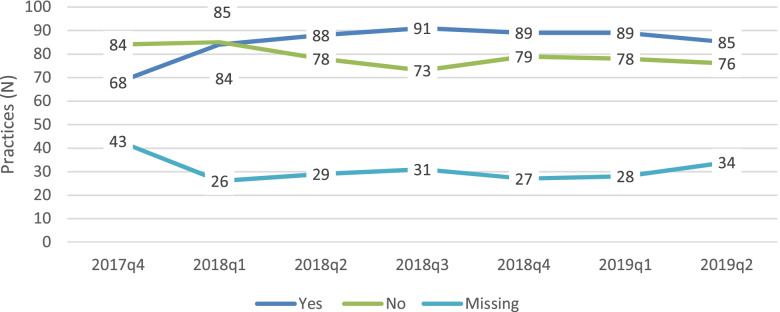
Engagement in additional bonus compensation over the intervention period. *Practice level (*N* = 195)

Of 177 practices entitled, 158 received ≥ one additional bonus payouts (89.3%). Regarding content of the additional compensation intervention, a mean of 51.8% of the maximum bonus size per index patient was achieved. Figure 4 illustrates the engagement in additional bonus compensation reimbursements on practice level and provides the number of practices where the bonus was triggered per quarter of the intervention period. For the first four quarters, an incline from 68 to 91 practices was observed which slightly dropped to 85 practices in quarter seven. Additional bonus payment was triggered for 11.3% (*N* = 22) of the practices in each of the seven quarters, for 17.9% (*N* = 35) it was triggered once and for 19% (*N* = 37) of practices it was never triggered. Additional File 2, Supplementary Table 3 provides the number of practices per number of quarters in which additional bonus payment was triggered.

### Participant views on implementation

Measures representing the five domains of participants’ views on implementation arose from the T2 questionnaire of the ARena survey study. At this time of measurement 63% (*N* = 184) of invited physicians responded of which 30.9% were female. Participants had a mean age of 54.2 years (SD = 7.9) and 26.4 years of occupational experience (SD = 7.9). Out of 14 included PCNs in the ARena study, every network was represented by two to 32 physicians. On practice level, one practice was represented by one to four physicians. Sample characteristics regarding intervention arm affiliations are demonstrated in Table 2.

**Table 2:** Sample characteristics of survey participants (T2)

**Characteristic**	**Arm I** (*N* = 68)	**Arm II** (*N* = 69)	**Arm III** (*N* = 49)	**Total** (*N* = 184)
Sex m % (n)	69.1 (47)	71.6 (48)	61.2 (30)	67.9 (125)
Age Mean (SD)	53.3 (6.8)	54.4 (8.4)	55.3 (7.5)	54.2 (7.6)
Experience Years Mean (SD)	25.7 (7.0)	26.8 (8.6)	27.0 (8.3)	26.4 (7.9)
# of members per PCN Median (Min/Max)	17 (11/23)	11 (5/32)	8 (2/18)	12 (2/32)
# of members per practice Median (Min/Max)	1 (1/4)	1 (1/3)	1 (1/4)	1 (1/4)

The reliability of items within scores varied between $\alpha$ = 0.423 and $\alpha$ = 0.914. The highest level of agreement was detected in the Context domain representing the role of PCNs in the ARena program (Mean = 4.1, SD = 0.8). The lowest level of agreement was identified in in the domain of positive attributions to AB prescribing (Mean = 2.6, SD = 0.9). Regarding multicollinearity of scores, correlations varied between *r* = 0.15 and *r* = 0.61. Comprehensive descriptive statistics of domain scores representing participants’ views on implementation are provided in Additional File 2, Supplementary Table 4.

### Participant views affecting key component engagement

Binary correlations between engagement in the additional bonus compensation scheme and participant views were identified in participant responsiveness (*r* = 0.399), context (*r* = 0.261) and quality of delivery (*r* = 0.170). The regression analyses showed significant effects of participant responsiveness, Context and intervention arm affiliation. The explanation of variance was determined at Nagelkerkes ${R}^{2}$ = 0.355. The correlation within clusters (subject = practices) was assessed by the intra class correlation coefficient (ICC) and was at ICC = 0.091. In the multilevel regression analysis, effect sizes stayed consistent but levels of significance in the domain of context as well as intervention arm affiliation merely decreased. Detailed estimates of parameters are provided in Table 3 and Additional File 2, Supplementary Table 5. Regarding engagement in the four offered QC themes, binary correlations were identified in domains of participant responsiveness (*r* = 0.508), Context (*r* = 0.351) and quality of delivery (*r* = 0.187). The regression analyses showed significant effects of in domains of participant responsiveness, context and culture of SDM. The adjusted R square of the multiple linear regression model was at ${R}^{2}$ = 0.299. Intra cluster correlations were determined at ICC = 0.16. In the additionally conducted multilevel model, deteriorations of effect sizes and levels of significance regarding context and culture of SDM were fractional. The effect sizes of participant responsiveness were marginally higher. Extensive reporting of estimates is provided in Table 4 and Additional File 2, Supplementary Table 6.

**Table 3:** Estimates regarding engagement in additional bonus compensation

	Odds Ratio	Lower CI limit	Upper CI limit	St error	*P*- value
Additional compensation engagement^*^					
Participant responsiveness	2.298	1.598	3.305	.185	.000
Quality of delivery	.668	.396	1.128	.267	.131
Context	2.146	1.135	4.055	.325	.019
Positive AB attribution	.870	.531	1.424	.252	.579
Culture of SDM	1.456	.700	3.029	.374	.314
Age	.972	.922	1.026	.027	.305
Sex (male)	.953	.397	2.285	.446	.913
Intervention Arm I (Constant)					
Arm II	.645	.251	1.659	.482	.363
Arm III	.272	.102	.725	.501	.009

^*^(*N* = 184 physicians); Nagelkerkes R Square = .355

**Table 4:** Estimates regarding engagement in QC themes

	Coef. B	Beta	Lower CI Limit	Upper CI Limit	St error	*P*- value
QC engagement^*^						
Participant responsiveness	.718	.470	.479	.957	.121	.000
Quality of delivery	-.178	-.140	-.397	.040	.110	.108
Context	.323	.201	.055	.590	.136	.019
Positive AB attribution	-.041	-.030	-.225	.144	.093	.664
Culture of SDM	-.334	-.172	-.614	-.053	.142	.020
Age	.018	.111	-.004	.040	.011	.108
Sex (male)	.129	.049	-.211	.470	.172	.455
Intervention Arm I (Constant)						
Arm II	.136	.053	-.246	.518	.193	.482
Arm III	-.102	-.038	-.495	.290	.199	.607

^*^(*N* = 184 physicians); adjusted R Square = .299

## Discussion

This study investigated the fidelity to the implementation program components QCs and additional bonus compensation, which were provided across all three ARena intervention arms. The overall fidelity in the quality improvement program in ARena was exceptionally high. This may be related to the particular setting of the ARena trial in PCNs, which was considered to be a supportive setting for the effort to promote rational antibiotic prescribing. For both, QCs and the additional bonus compensation program, a positive attribution to PCN membership was a promoting force of intervention engagement. A previous qualitative study conducted within the ARena PE identified various factors that may have contributed to these impacts [39]. Particularly, peer exchange opportunities, social support, promotion of self-reflection and knowledge manifestation were accelerators of care improvement.

Focusing on participants’ engagement in QC themes, highest attendance rates were measured in CRTI themes and followed by UTI and CAP subjects. QCs regarding MRP issues were least visited as this issue may not be as relevant for the outpatient care setting. Although attendance rates in ARena can be considered high, they were noticeably lower than observed in previously conducted QC initiatives targeting prescribing patterns of primary care physicians in Germany [28] which may be owed to mechanisms of bundled interventions. Established QC movements have shown to be a facilitator to the engagement in this intervention [23]. The German National Association of Statutory Health Insurance Physicians reported in 2018 that 8 400 QCs were conducted in outpatient care [43]. Since these observations indicate familiarity of German resident physicians with this delivery mode of quality improvement initiatives, it presumably has been a determinant of explanation for high QC attendance rates observed in ARena. However, research points to unstandardized procedures of this complex intervention which still impedes high quality recommendations on the effectiveness of QCs [25]. Regarding benchmarking procedures provided in QC sessions, results of a cluster RCT indicate that e-mails providing comparison of antibiotic prescribing rates with local peers did provide small effect sizes to declined prescribing rates [44]. Therefore, a provision of benchmarking data via email may be a convenient approach to reduce participation hurdles and to further tailor interventions. Contrary to results of a SDM culture being an impeding influence to the engagement in QC themes, positive attributions of SDM on rational antibiotic prescribing patterns itself are referred in a systematic review [45].

Considering exposure to additional bonus compensation, 88.4% of practices received compensation at least one time for appropriate prescribing. However, during the seven quarters in which the additional bonus was offered, only 11.3% of practices triggered the payment continuously, which is surprising in light of the view that financial compensation is required for quality improvement (dominant in some policy debates). Explanations of the inconsistency in engagement in additional compensation over the intervention period did not emerge from the available data. Findings in the PE conducted alongside ARena indicate that interviewed physicians did interpret additional compensation as one key to generate behavior change. However, additional compensation might only be an incentive to participate in a study, but of lesser importance after this decision is taken [41]. Jan et al. [46] also identified increased administrative workloads and inadequate understandings of performance-based payment contents as principal reasons for aversions of family practitioners to engage. These aspects are not echoed in this study but can be considered for explanation. Besides, the likelihood of additional bonus payment programs to show desirable effects are reported to be three times higher with larger incentives [47]. Since reimbursements in ARena were proportionately small, this may also explain heterogeneity. Generally, research indicates that German physicians widely voice concerns about establishing additional bonus compensation since they apprehend ethical conflicts between monetary interests and patients’ safety, imposed autonomy of the medical profession as well as a loss of autonomy for the benefit of statutory health insurance funds [48]. Therefore, it could be beneficial to develop additional bonus compensation programs for primary care with theory-driven framework approaches in which needs of target groups are accounted [49].

Previously conducted fidelity analyses of multifaceted effectiveness studies were heterogeneous in theory and designs. For instance, a back pain prevention program among nursing aides introduced in a stepped-wedge trial, was evaluated by a quantified, theory-driven approach using logbooks as well as one-time questionnaires and focused on domains of participation, exposure and responsiveness [50]. Notably, this study considered the evaluation of fidelity to be a domain of separated interest but not as a subordinated construct of listed domains. In a health promotion study fostering physical activity of patients in Dutch rehabilitation centers, implementation fidelity was assessed by a longitudinal survey design in order to detect time trends of fidelity scores [51]. Accompanied by qualitative data collection, organizational and professional differences between clustered centers were explored. A further quantitative fidelity analysis utilized attendance lists, checklists, worksheets, exit surveys and expert observations to allow a sophisticated view on a team-based, behavioral intervention in care aids [52]. Focusing on rational antibiotic prescribing efforts, research on antimicrobial stewardship (AMS) programs in British community healthcare organizations was applied by a web-based survey strategy to gain insights about the engagement in introduced stewardship toolkits being a part of a five-year cross-governmental awareness strategy [53]. In a cluster randomized controlled trial which aimed to promote AMS among British community pharmacies to improve the management of respiratory tract infections, an accompanied process evaluation was conducted [54] in a cross-sectional survey design based on the COM-B (capability, opportunity, motivation and behavior) model [55].

The sketch of previously conducted quantitative fidelity studies highlights the need of tailored concepts which meet specific conditions of respective implementation programs. Portrayed programs were most commonly limited to descriptive score analyses and in some cases extended to mixed model procedures which examined the variance of primary trial outcomes between clusters. Thematically, fidelity analyses of programs regarding a rational antibiotic use were scarce. Investigations considering a dose–response-relationship between the engagement in specific interventions and primary study outcomes have not been identified. Nevertheless, statistical modelling between intervention dosage and primary outcome response appears to be beneficial to detect not only fidelity to study protocols, but to adopt programs so that best possible effect sizes can be achieved. To attain this goal, a discussion about standardized theoretical conceptualization and appropriate data sources may be useful. On the basis of this study and referenced approaches, a mix of methods containing attendance data, self-reports and qualitative investigations seems necessary to extensively examine the theoretically driven concept of fidelity and to offer practical recommendations for future implementation adjustments.

### Strengths and limitations

This study strengthens the appraisal of the ARena program regarding intervention fidelity and feasibility of implementation. A profound theoretical conceptualization offered chances to quantify influences of participant views on implementation with intervention engagement. Such approaches guide further developments of programs and allow opportunities for adaptation. The combination of attendance with survey data ensured data triangulation and guided a holistic view on fidelity of core components in the ARena program.

Some limitations must be reported. Initially, it was intended to match data of the present fidelity analysis with data of the primary outcome analysis on practice level as this was expected to potentially generate additional insights into favorable dosages of intervention components in order to achieve best possible outcomes. However, German data protection law did not allow for this type of data linkage. Engagement data used for regression modelling originated from self-reported survey data which implies the risk of social desirability bias. Since this study was explorative in design, insecurities regarding construct validity of calculated scores reflecting domains of participant views and statistical power of sample sizes remain. Moreover, the absence of qualitative data integration prevents explanations of some results. Reasons for low levels of bonus size achievements were not observed. Noticeably, the term of fidelity implies understandings of a ‘gold-standard’ in program implementation. This can be misleading, since adjustments must be considered reasonable to achieve the best possible results under real life conditions and thus must be respected in final assessments.

## Conclusion

This study explored the intervention fidelity of a complex intervention in real-world healthcare practice. The linkage of reported engagement with intervention components with perceptions of domains of views on implementation facilitates explanation of the variation of effects and contributes to the development of tailored implementation programs in the future. The fidelity analysis of this study indicates a robust feasibility of the completed ARena implementation program and the overall fidelity to it, in particular regarding QC and bonus compensation components which represented core elements of this complex intervention. The engagement in study components was facilitated by positive attributions towards PCN memberships and responsiveness to interventions. In QCs, efforts of SDM antagonized intervention engagement. These insights support further tailoring efforts of complex interventions in the context of rational antibiotic use in German ambulatory care. Future investigations should consider dose response calculations to adapt frequencies regarding exposure to interventions. In prospect, efforts for standardized quantitative fidelity analyses will be conducive to support a holistic view on this concept as well as comparability of evaluations.

## Supplementary Information

**Additional file 1: Supplementary Figure 1.** Study Design & participant numbers of the ARena trial.

**Additional file 2: Supplementary Table 1.** Survey items (T2) included for scores of participant views (*N* = 184).** Supplementary Table 2.** Indicators reflecting fidelity to the ARena program (*N* = 290 physicians).** Supplementary Table 3.** Number of quarters with claimed P4P reimbursements (*N* = 195 practices).** Supplementary Table 4.** Scores of domains reflecting participant views (T2) (*N* = 184 physicians).** Supplementary Table 5**. Multilevel logistic regression model clustered by practice affiliation regarding use of the bonus payment component (*N* = 184 physicians).** Supplementary Table 6.** Multilevel multiple linear regression model clustered by practice affiliation regarding attendance to QC themes (*N* = 184).

## Data Availability

Due to data protection regulations by the German law and the data provider, original data sets cannot be made accessible. All analyses conducted in this study are provided in the Additional File 1.

## Abbreviations

AB: Antibiotic
abc: Additional bonus compensation
AMS: Antimicrobial stewardship
AOK: Public organization of statutory health insurance
ARena: Sustainable reduction of antibiotic-induced antimicrobial resistance
AUI: Acute uncomplicated infections
CAP: Community acquired pneumonia
CDSS: Computerized decision support system
CRTI: Common respiratory tract infections
ICC: Intra class coefficient
IQR: Inter quartile range
MA: Medical assistant
MCAR: Missing Completely at Random
MRP: Multi-resistant pathogens
OR: Odds Ratio
PCN: Primary care network
PE: Process evaluation
SDM: Shared decision-making
UTI: Urinary tract infection
QC: Quality circle
Q1: Quarter one
Q2: Quarter two
