# Supplementary material for: Implementation fidelity in a multifaceted program to foster rational antibiotics use in primary care: an observational study
Source: BMC Med Res Methodol. 2022 Sep 19;22:243. doi: 10.1186/s12874-022-01725-3 (PMC9487096; doi:10.1186/s12874-022-01725-3)
Supplement: Supplementary file 1 — Additional file 1: Supplementary Figure 1. Study Design & participant numbers of the ARena trial. [file 12874_2022_1725_MOESM1_ESM.docx]

# Additional File 1, Supplementary Figure 1:


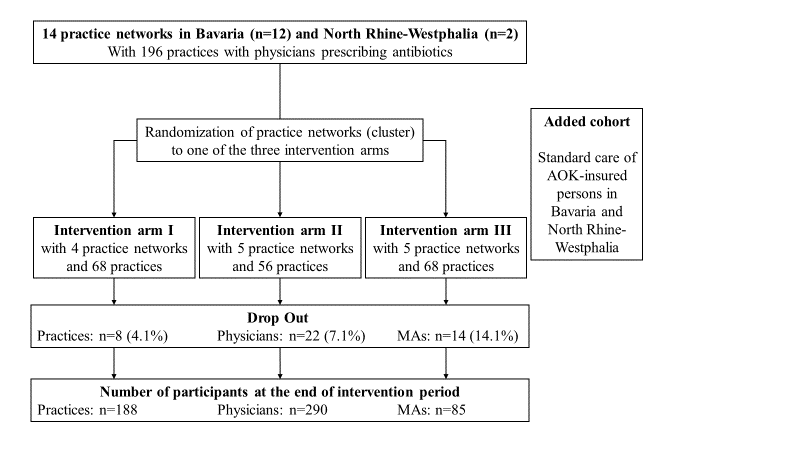


**Supplementary Figure 1**: Study Design & participant numbers of the ARena trial
