# Supplementary material for: Implementation fidelity in a multifaceted program to foster rational antibiotics use in primary care: an observational study
Source: BMC Med Res Methodol. 2022 Sep 19;22:243. doi: 10.1186/s12874-022-01725-3 (PMC9487096; doi:10.1186/s12874-022-01725-3)
Supplement: Supplementary file 2 — Additional file 2: Supplementary Table 1. Survey items (T2) included for scores of participant views (N = 184). Supplementary Table 2. Indicators reflecting fidelity to the ARena program (N = 290 physicians). Supplementary Table 3. Number of quarters with claimed P4P reimbursements (N = 195 practices). Supplementary Table 4. Scores of domains reflecting participant views (T2) (N = 184 physicians). Supplementary Table 5. Multilevel logistic regression model clustered by practice affiliation regarding use of the bonus payment component (N = 184 physicians). Supplementary Table 6. Multilevel multiple linear regression model clustered by practice affiliation regarding attendance to QC themes (N = 184). [file 12874_2022_1725_MOESM2_ESM.pdf]

Additional File 2, Supplementary Tables 1-6:

Supplementary Table 1: Survey items (T2) included for scores of participant views (N = 184)

Participant views on implementation	Name	Description	Value	Scale	Cronbachs Alpha
Participant responsiveness	qz_new_imp	*Quality circles provided new impulses*	1 = Strongly disagree<br>2 = Disagree<br>3 = Neutral<br>4 = Agree<br>5 = Strongly Agree	metric	0.749
	qz_new_integr	*Newly gained knowledge was integrated into practice routines*	1 = Strongly disagree<br>2 = Disagree<br>3 = Neutral<br>4 = Agree<br>5 = Strongly Agree	metric	
	qz_reflect	*Quality circles stimulated the reflection of routines*	1 = Strongly disagree<br>2 = Disagree<br>3 = Neutral<br>4 = Agree<br>5 = Strongly Agree	metric	
	qz_effort	*Realization of QC content was associated with great effort*	1 = Strongly disagree<br>2 = Disagree<br>3 = Neutral<br>4 = Agree<br>5 = Strongly Agree	metric	
Participant Responsiveness	abc_new_imp	*Additional bonus compensation provided new impulses*	1 = Strongly disagree<br>2 = Disagree<br>3 = Neutral<br>4 = Agree<br>5 = Strongly Agree	metric	0.914
	abc_reflect	*Additional bonus compensation stimulated the reflection of routines*	1 = Strongly disagree<br>2 = Disagree<br>3 = Neutral<br>4 = Agree<br>5 = Strongly Agree	metric	
Quality of Delivery	moti_guideline	*ARena participation motivates to treat patients guideline-oriented*	1 = Strongly disagree<br>2 = Disagree<br>3 = Neutral<br>4 = Agree<br>5 = Strongly Agree	metric	0.849
	change_strategy	*ARena participation changed antibiotic-prescribing strategy*	1 = Strongly disagree<br>2 = Disagree<br>3 = Neutral<br>4 = Agree<br>5 = Strongly Agree	metric	
	secure_decision	*ARena participation provided safety in therapeutic decision*	1 = Strongly disagree<br>2 = Disagree<br>3 = Neutral<br>4 = Agree<br>5 = Strongly Agree	metric	
Context	support_implem	*PCN supports the implementation of ARena interventions*	1 = Strongly disagree<br>2 = Disagree<br>3 = Neutral<br>4 = Agree	metric	0.885

			5 = Strongly Agree		
	support_integr	*PCN supports integration of newly learned information*	1 = Strongly disagree<br>2 = Disagree<br>3 = Neutral<br>4 = Agree<br>5 = Strongly Agree	metric	
	network_imp_new	*PCN provided new impulses for patient care*	1 = Strongly disagree<br>2 = Disagree<br>3 = Neutral<br>4 = Agree<br>5 = Strongly Agree	metric	
	network_refl_rou	*PCN encouraged the reflection of previous routines*	1 = Strongly disagree<br>2 = Disagree<br>3 = Neutral<br>4 = Agree<br>5 = Strongly Agree	metric	
Culture of SDM	dec_fact_pref	*Patient desires are considered in decisions regarding antibiotic prescribing*	1 = Strongly disagree<br>2 = Disagree<br>3 = Neutral<br>4 = Agree<br>5 = Strongly Agree	metric	0.423
	dec_fact_peer	*Peer exchange is considered in decisions regarding antibiotic prescribing*	1 = Strongly disagree<br>2 = Disagree<br>3 = Neutral<br>4 = Agree<br>5 = Strongly Agree	metric	
	dec_fact_exp	*Previous experiences are considered in decisions regarding antibiotic prescribing*	1 = Strongly disagree<br>2 = Disagree<br>3 = Neutral<br>4 = Agree<br>5 = Strongly Agree	metric	
Positive AB attribution	pat_happy	*Antibiotic prescribing leads to increased patient satisfaction*	1 = Strongly disagree<br>2 = Disagree<br>3 = Neutral<br>4 = Agree<br>5 = Strongly Agree	metric	0.674
	pat_freq	*Antibiotic prescribing leads to a decline in consultation frequency*	1 = Strongly disagree<br>2 = Disagree<br>3 = Neutral<br>4 = Agree<br>5 = Strongly Agree	metric	

Supplementary Table 2: Indicators reflecting fidelity to the ARena program (N = 290 physicians)

**Domain**	**Indicator**	**Numerator**	**Denominator**	**Value** Ratio (%)
Exposure	Execution physician QCs	Conducted QCs	Scheduled QCs	54/56 (96.4)
Exposure	compensation reimbursement	Practices receiving ≥ 1 reimbursement	Practices entitled to receive reimbursement	177/158 (89.3)
Content	Level of bonus size achievement	Mean bonus size per index patient	Maximum bonus size per index patient	2.59€/5.00€ (51.8)
Engagement	Continuous ARena participation	Number of physicians who continuously participated in ARena	Number of ARena physicians	297/318* (93.4)
Engagement	additional compensation	Participants who received compensation ≥ 1 patient case	Number of potentially active participants	244/276 (88.4)
Engagement	Attendance rate physician QCs	Number of attendees (all 4 QC topics)	Number of potentially active participants	645/1150 (56.1)
Engagement	Attendance rate e-learning	Number of attendees who finished e-learning	Number of ARena physicians	282/318* (88.7)
Engagement	Basic expenditure reimbursement claimed	Number of physicians claiming monetary expenditure	Number of ARena physicians	249/318* (78.3)

*Physicians working in several practices counted in each practice.

Supplementary Table 3: Number of quarters with claimed P4P reimbursements (N = 195 practices)

**Claimed P4P** reimbursements	0	1	2	3	4	5	6	7
# practices	37	35	17	25	21	16	22	22
Percentage %	19.0	17.9	8.7	12.8	10.8	8.2	11.3	11.3
Cumulative %	19.0	36.9	45.6	58.5	69.2	77.4	88.7	100

Supplementary Table 4: Scores of domains reflecting participant views (T2) (N = 184 physicians)

Score	N	Min	Max	Mean	SD	Cronbachs Alpha
Participant responsiveness QCs	175	1	5	3.8	0.8	0.749
Participant responsiveness compensation	163	1	5	2.8	1.3	0.914
Quality of delivery improvements	181	1	5	3.8	1.0	0.849
Contextual facilitators (PCN)	182	1	5	4.1	0.8	0.885
Positive AB attribution	182	1	5	2.6	0.9	0.674
Culture of SDM	183	1	5	3.4	0.6	0.423

Supplementary Table 5: Multilevel logistic regression model clustered by practice affiliation regarding use of bonus payment component (N = 184 physicians)

	Odds Ratio	Lower CI limit (Wald)	Upper CI limit (Wald)	St. error	p-value
**utilization**					
Participant responsiveness	2.298	.474	1.190	.183	.000
Quality of delivery	.668	-.971	.164	.290	.164
Context	2.146	.105	1.422	.336	.023
Positive AB attribution	.870	-.695	.416	.284	.623
Culture of SDM	1.456	-.488	1.240	.441	.394
Age	.972	-.088	.032	.031	.363
Sex (male)	.953	-.961	.864	.466	.917
Intervention Arm A (Constant)					
Arm B	.645	-1.296	.419	.437	.316
Arm C	.272	-2.289	-.317	.503	.010

Supplementary Table 6: Multilevel multiple linear regression model clustered by practice affiliation regarding attendance to QC themes (N = 184)

	Coefficient B	Lower CI limit	Upper CI limit	St. error	p-value
**QC utilization**					
Participant responsiveness	.723	.492	.954	.117	.000
Quality of delivery	-.178	-.389	.034	.107	.099
Context	.317	.057	.577	.131	.017
Positive AB attribution	-.041	-.220	.137	.090	.644
Culture of SDM	-.333	-.604	-.061	.137	.017
Age	.017	-.004	.038	.011	.113
Sex (male)	-.139	-.469	.190	.167	.406
Intervention Arm A (Constant)					
Arm B	-.136	-.511	.238	.189	.473
Arm C	.092	-.294	.479	.195	.637
